# Modeling complement activation on human glomerular microvascular endothelial cells

**DOI:** 10.3389/fimmu.2023.1206409

**Published:** 2023-10-25

**Authors:** Kes H. Stevens, Laura M. Baas, Thea J. A. M. van der Velden, Romy N. Bouwmeester, Niels van Dillen, Eiske M. Dorresteijn, Arjan D. van Zuilen, Jack F. M. Wetzels, Marloes A. H. M. Michels, Nicole C. A. J. van de Kar, Lambertus P. van den Heuvel

**Affiliations:** ^1^ Department of Pediatric Nephrology, Amalia Children’s Hospital, Radboud University Medical Center, Nijmegen, Netherlands; ^2^ Department of Pediatric Nephrology, Sophia Children’s Hospital, Erasmus Medical Center, Rotterdam, Netherlands; ^3^ Department of Nephrology and Hypertension, University Medical Center Utrecht, Utrecht, Netherlands; ^4^ Department of Nephrology, Radboud University Medical Center, Nijmegen, Netherlands; ^5^ Department of Laboratory Medicine, Radboud University Medical Center, Nijmegen, Netherlands; ^6^ Department of Pediatrics/Pediatric Nephrology, University Hospitals Leuven, Leuven, Belgium; ^7^ Department of Development and Regeneration, University Hospitals Leuven, Leuven, Belgium

**Keywords:** alternative pathway, atypical hemolytic uremic syndrome, C5b-9, complement system, eculizumab, glomerular endothelium

## Abstract

**Introduction:**

Atypical hemolytic uremic syndrome (aHUS) is a rare kidney disease caused by dysregulation of the complement alternative pathway. The complement dysregulation specifically leads to damage to the glomerular endothelium. To further understand aHUS pathophysiology, we validated an *ex vivo* model for measuring complement deposition on both control and patient human glomerular microvascular endothelial cells (GMVECs).

**Methods:**

Endothelial cells were incubated with human test sera and stained with an anti-C5b-9 antibody to visualize and quantify complement depositions on the cells with immunofluorescence microscopy.

**Results:**

First, we showed that zymosan-activated sera resulted in increased endothelial C5b-9 depositions compared to normal human serum (NHS). The levels of C5b-9 depositions were similar between conditionally immortalized (ci)GMVECs and primary control GMVECs. The protocol with ciGMVECs was further validated and we additionally generated ciGMVECs from an aHUS patient. The increased C5b-9 deposition on control ciGMVECs by zymosan-activated serum could be dose-dependently inhibited by adding the C5 inhibitor eculizumab. Next, sera from five aHUS patients were tested on control ciGMVECs. Sera from acute disease phases of all patients showed increased endothelial C5b-9 deposition levels compared to NHS. The remission samples showed normalized C5b-9 depositions, whether remission was reached with or without complement blockage by eculizumab. We also monitored the glomerular endothelial complement deposition of an aHUS patient with a hybrid *complement factor H (CFH)/CFH-related 1* gene during follow-up. This patient had already chronic kidney failure and an ongoing deterioration of kidney function despite absence of markers indicating an aHUS flare. Increased C5b-9 depositions on ciGMVECs were observed in all samples obtained throughout different diseases phases, except for the samples with eculizumab levels above target. We then tested the samples on the patient’s own ciGMVECs. The C5b-9 deposition pattern was comparable and these aHUS patient ciGMVECs also responded similar to NHS as control ciGMVECs.

**Discussion:**

In conclusion, we demonstrate a robust and reliable model to adequately measure C5b-9-based complement deposition on human control and patient ciGMVECs. This model can be used to study the pathophysiological mechanisms of aHUS or other diseases associated with endothelial complement activation *ex vivo*.

## 1 Introduction

Atypical hemolytic uremic syndrome (aHUS) is a rare but severe kidney disease belonging to the group of thrombotic microangiopathies (TMA) and is characterized by mechanical hemolytic anemia, thrombocytopenia and acute kidney injury (AKI) (1, 2). The damage to the glomerular endothelium is caused by a dysregulation of the alternative pathway (AP) of the complement system. Before the introduction of the complement inhibitor eculizumab, around 50% of the aHUS patients progressed into end-stage kidney failure, and mortality rates up to 25% in the acute phase were reported (3–8). With eculizumab, a humanized monoclonal antibody targeting C5, morbidity and mortality rates have significantly improved (9, 10).

The complement system is a powerful tool of the innate immune system to eliminate pathogens and apoptotic/necrotic host cells. The system can be initiated via three different pathways: the classical pathway, lectin pathway, and AP (11–13). In contrast to the classical and lectin pathway, the AP is constitutively active at a low level by a process called ‘tick-over’. This results in low levels of active C3, able to form C3 convertases that cleave C3 into C3a and C3b. C3b is an important opsonin, but also supports subsequent formation of C5 convertases that cleave C5 into C5a and C5b. C5a is a powerful chemoattractant, and C5b supports formation of the C5b-9 complex, also known as the membrane attack complex. C5b-9 formation on the cell leads to impaired membrane integrity and serious cell damage (14). Therefore, the complement system, and especially the AP, is strictly regulated in physiological state to prevent host cell damage.

In up to 75% of the aHUS patients, genetic variants in genes encoding complement components are found (15–18). The most common are loss-of-function mutations in genes encoding for the complement regulatory proteins factor H (FH), factor I and membrane-cofactor protein (MCP), and gain-of-function mutations in complement C3 and factor B. In addition, genomic rearrangements in the *complement FH (CFH)* and *CFH related (CFHR) 1-5* region have been described, resulting in hybrid FH-FHR proteins which may interfere with normal complement regulation (19, 20). Furthermore, autoantibodies against FH may impair the homeostatic complement regulation and lead to aHUS (21). The disease penetrance of aHUS in mutation carriers is incomplete. Various triggering factors, such as infections, pregnancy, and/or certain medications, can elicit the development of aHUS (17, 22, 23).

Especially the kidneys are vulnerable for complement deposition and the subsequent TMA-mediated injury (24, 25). The glomerular microvascular endothelial cells (GMVECs) are the primary target of AP activation in aHUS, but the exact mechanisms behind this high susceptibility of GMVECs to complement deposition remains unknown. A possible explanation might be their morphological (fenestration) and functional (filtration) difference compared to other endothelial cells (25, 26).

Nonetheless, in the last decade, other endothelial cell types than GMVECs have frequently been used to study complement activation and deposition in patients with complement-mediated kidney diseases. For example, human dermal microvascular endothelial cells 1 (HMECs-1) (27–31) and human umbilical vein endothelial cells (HUVECs) (32–34) have been described as cell models for aHUS. However, these cells might not optimally reflect the *in vivo* situation, given the specific glomerular cell vulnerability in aHUS. Therefore, the aim of this study was to model complement activation in aHUS patients *ex vivo*, by measuring complement deposition on human conditionally immortalized GMVECs (ciGMVECs). Moreover, by having the unique possibility to create a ciGMVEC line derived from an aHUS patient, we could model the complement activation in samples of an aHUS patient on patient’s own endothelial cells.

## 2 Materials and methods

### 2.1 Ethics

Written informed consent was obtained from all aHUS patients or their legal guardians, and from all healthy controls of whom blood samples were used in this study. Informed consent was also obtained for isolating GMVECs from an aHUS patient (P1) who had bilateral nephrectomy in preparation to kidney transplantation. The study was performed in accordance with the appropriate version of the Declaration of Helsinki.

### 2.2 aHUS patients

Six patients diagnosed with aHUS were included in this study and were genetically characterized (**Table 1**). P1 was identified with a heterozygous deletion of *CFH* exon 22, *CFHR3* and *CFHR1* exon 1-5, indicating a genomic rearrangement resulting in a *CFH/CFHR1* hybrid gene (35). This rearrangement has been described to lead to a severe aHUS phenotype (36). P2 and P4-P6 carried a pathogenic *C3* variant, i.e. c.481C>T (p.Arg161Trp), resulting in decreased binding to FH and MCP, and increased binding to complement factor B, leading to a C3 convertase with increased activity (34, 37, 38). In P3, a homozygous *CFHR3-1* deletion was found in combination with autoantibodies against FH (39–41). P3 (P4 in Bouwmeester et al.), P4 (P12 in Bouwmeester et al.), and P5 (P18 in Bouwmeester et al.) were described in the CUREiHUS study (18). All patients were treated according to a restrictive eculizumab protocol, as described in the CUREiHUS study (18, 43).

**Table 1:** Genetics of atypical hemolytic uremic syndrome (aHUS) patients.

Patient	Gene	cDNA change	Protein change	Zygosity	Class^a^	FH autoantibodies	References
P1	*CFH-CFHR1* hybrid^b^			Heterozygous		Negative	(35, 36)
P2	*C3*	c.481C>T	p.Arg161Trp	Heterozygous	5	Negative	(34, 37, 38)
P3	*CFHR3-1* deletion			Homozygous	2	Positive	(39–41)
P4	*C3*	c.481C>T	p.Arg161Trp	Heterozygous	5	Negative	(34, 37, 38)
P5	*C3*	c.481C>T	p.Arg161Trp	Heterozygous	5	Negative	(34, 37, 38)
P6	*C3*	c.481C>T	p.Arg161Trp	Heterozygous	5	Negative	(34, 37, 38)

a Classification based on guidelines of the American College of Medical and Genomics (42) as benign (class 1), likely benign (class 2), uncertain significance (class 3), likely pathogenic (class 4), and pathogenic (class 5).

b Genomic analysis using multiplex ligation-dependent probe amplification identified a heterozygous deletion of *CFH* (LRG_47t1) exon 22, complete *CFHR3* (LRG_175t1) and CFHR1 (LRG_149t1) exon 1-5, indicating a genomic rearrangement resulting in a *CFH/CFHR1* hybrid gene (NC_000001.10: g.(196715167_196716320)_(196799888_196801005)del).

*CFH, complement factor H; CFHR, complement factor H related.*

P1 is known with aHUS from the age of 5 months and treated until the age of 11 years with chronic plasma therapy. He then was successfully switched to treatment with eculizumab. At the age of 15 years a further deterioration of his kidney function was observed without any signs of recurrence of aHUS. His radiographic examinations revealed an acquired glomerulocystic disease, a reduced left kidney function, and an abnormal venous system of unknown origin. A bilateral nephrectomy was performed to minimize the risk for aHUS recurrence after kidney transplantation. The blood taken at the age of 15 years and used in this study was marked as day 0 (d0). The medical history of this aHUS patient (P1) was in depth studied and reported previously (36).

TMA was defined by ≥2 of the following criteria: thrombocytopenia (platelet count <150 ×10^9^/l), lactate dehydrogenase (LDH) above the upper limit of normal (>250 U/l) and low/undetectable haptoglobin (<0.3 mg/l). AKI was defined as an increase in serum creatinine of ≥26.53 µmol/l (0.3 mg/dl) in 48 hours or ≥1.5 times baseline in <7 days.

### 2.3 Cell culture

GMVECs of P1 were isolated from kidney tissue obtained after nephrectomy and subsequently conditionally immortalized as described by Satchell et al. (44). Control ciGMVECs (obtained from Satchell et al. (44)) and P1 ciGMVECs were allowed to proliferate by culturing them at 33°C with 5% (v/v) CO_2_ on 96-well plates (655090, Greiner, Cellstar^®^) coated with gelatin (48720, FLUKA). When confluent, cells were transferred to 37°C with 5% (v/v) CO_2_ to induce growth arrest. After 7-8 days, cells were used for experiments. The growth medium, later referred to as endothelial medium, consisted of M199 medium (22340, Gibco) supplemented with 10% (v/v) heat-inactivated (HI) human serum (ISER, Innovative research), 10% (v/v) HI newborn calf serum (26010, Gibco), 2 mM L-glutamine (25030, Gibco), 100 U/ml penicillin, 100 mg/ml streptomycin (15140, Gibco), 5 U/ml heparin (Leo Pharmaceuticals), and 150 µg/ml bovine endothelial growth factor made according to the protocol described by Maciag et al. (45).

Primary GMVECs from human origin were obtained from kidney tissue after nephrectomy and cultured as previously described (46). Primary GMVECs were cultured at 37°C with 5% (v/v) CO_2_ on gelatin-coated 96-well plates with endothelial medium until confluent.

HMECs-1 (ATCC^®^ CRL3243™) were cultured at 37°C with 5% (v/v) CO_2_ on uncoated 96-well plates with MCDB 131 medium (10372, Gibco) supplemented with 50 µg/ml bovine endothelial growth factor, 10% (v/v) fetal calf serum (FCS; 10270, Gibco), 2 mM L-glutamine, 1 µg/ml hydrocortisone (H0888, Sigma-Aldrich), 100 U/ml penicillin, and 100 mg/ml streptomycin.

### 2.4 Sample collection and sample preparation

Blood samples of aHUS patients and healthy individuals were collected and processed as described previously (47). Pooled normal human serum (NHS) was obtained by pooling 10 or 14 healthy individual samples. To obtain HI-NHS, NHS was incubated for 30 minutes at 56°C. Samples were aliquoted and stored at -80°C until use.

Serum samples were thawed on ice and diluted to the desired serum concentration in Hanks’ balanced salt solution containing calcium and magnesium (HBSS++, 14025; Gibco) supplemented with 0.5% (w/v) bovine serum albumin (BSA; 820451, Millipore), later referred to as test medium. Where indicated, sera were pre-incubated for 5-10 minutes at room temperature with 300 µg/ml of the C5 blocker eculizumab (Soliris^®^, Alexion Pharmaceuticals) and/or 0.3 mg/ml zymosan (Z4250, Sigma-Aldrich). Zymosan was pre-diluted in magnesium-ethylene glycol tetra acetic acid buffer (Mg-EGTA; 2.03 mM veronal buffer, pH 7.4, 10 mM EGTA, 7 mM MgCl_2_, 0.083% gelatin, 115 D-glucose, and 60 mM NaCl). Eculizumab and zymosan concentrations are indicated per volume of undiluted serum.

### 2.5 Complement deposition and immunofluorescence staining

Cells cultured on 96-well plates were washed twice with test medium and pre-incubated with test medium for 15 minutes at 37°C with 5% (v/v) CO_2_. Next, cells were incubated with 33.3% serum in test medium with a final volume of 140 µl at 37°C with 5% (v/v) CO_2_ for 2 hours, unless stated otherwise. After incubation, cells were washed twice with HBSS++, fixed with 3% paraformaldehyde/phosphate buffered saline (PBS; 10010, Gibco) for 10 minutes, and washed twice with PBS.

For immunofluorescence staining, cells were first blocked with 2% (w/v) BSA/PBS for 1 hour. Then, cells were stained with mouse anti-CD31 (1:100, 555444, BD Pharmingen) and/or rabbit anti-C5b-9 (1:200; 204903, Calbiochem), diluted in 1% (w/v) BSA/PBS with 10% (v/v) goat serum (ab7481, Abcam), for 1 hour. After washing three times, cells were incubated with the secondary antibodies (1:1000 in 1% BSA/PBS) Alexa488-conjugated goat anti-rabbit (A11008, Invitrogen), Alexa568-conjugated goat anti-mouse (ab175473, Abcam), and/or FITC-conjugated rabbit anti-C3c (1:20; F0201, Dako; recognizes the C3c part of C3, C3b and iC3b), for 1 hour. Cell nuclei were additionally stained with 5 µg/ml 4’, 6-diamidino-2-phenylindole (DAPI; D1306, Invitrogen) for 5 minutes. All steps were performed with static conditions at room temperature. Images were captured with Zeiss Axio Observer 7 with artificial intelligence sample finder in combination with ZEN imaging software (Zeiss).

### 2.6 Quantification of C5b-9 deposition

For the quantification of C5b-9 depositions, an area of 4 mm^2^ of each well was visualized, with focusing on DAPI and with equal visualization settings for all experiments. Fluorescence images were analyzed utilizing Fiji 1.51n software with a custom-made ImageJ macro to quantify C5b-9 (48).

### 2.7 Flow cytometry

Control ciGMVECs in 24-well plates (3524, Costar^®^, Corning Incorporated) were washed twice with test medium and subsequently incubated with 10% (v/v) serum in test medium with a final volume of 280 µl at 37°C with 5% (v/v) CO_2_ for 1 hour. Next, cells were washed twice with HBSS++ and fixed with 0.5% paraformaldehyde/PBS for 15 minutes. After fixation, cells were stained with rabbit anti-C5b-9 (1:100) in 0.1% (v/v) BSA/PBS at 4°C for 30 minutes. After three washes with PBS, cells were incubated with Alexa488-conjugated goat anti-rabbit (1:100) at 4°C for 30 minutes. Cells were detached with 0.05% trypsin-ethylenediamine tetra acetic acid (25300, Gibco) and dissolved in 10% (v/v) FCS/PBS. Cells were spun down and resuspended in 0.1% (v/v) BSA/PBS. C5b-9 deposition was quantified using a CytoFLEX flow cytometer (Beckman Coulter) and results were analyzed with Kaluza 2.1.3 software (Beckman Coulter).

### 2.8 Collection of clinical data and standard laboratory measurement

Clinical and standard laboratory data were obtained from patients’ electronic medical records. Serum C5 levels were measured with enzyme-linked immunosorbent assay (ELISA) as described previously (49). Patients were tested for autoantibodies against FH using an in-house ELISA (50).

### 2.9 Statistical analysis

Data are expressed as mean ± standard deviation. All statistical analyses were performed with GraphPad Prism version 9.0.0 for Windows (GraphPad Software, San Diego, California, USA). Data were analyzed by unpaired t-test, one-way ANOVA, or two-way ANOVA, followed by *post-hoc* analysis as indicated in figure legends. A p-value (P) of <0.05 was considered as statistically significant.

## 3 Results

### 3.1 Comparing complement C5b-9 deposition on glomerular endothelial cells

**Figure 1:**
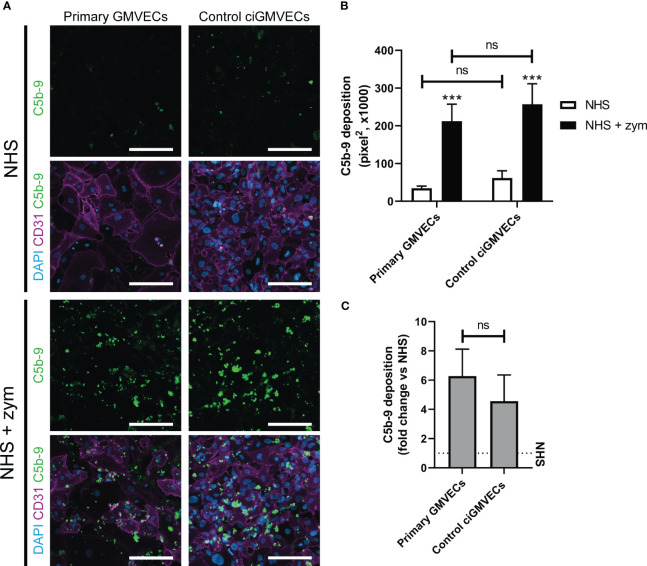
Serum-induced C5b-9 deposition on primary glomerular microvascular endothelial cells (GMVECs) and conditionally immortalized GMVECs (ciGMVECs). **(A-C)** Primary GMVECs and control ciGMVECs, from human origin, after incubation with 33.3% serum in test medium for 2 hours. **(A)** Representative immunofluorescence images of C5b-9 (green), CD31 (purple) and cell nuclei with 4’, 6-diamidino-2-phenylindole (DAPI; blue) after incubation with normal human serum (NHS) and NHS-zymosan (zym). Scale bar: 200 µm. **(B, C)** Quantification of C5b-9 deposition in pixel^2^ after serum incubation with NHS and NHS-zym **(B)** and as fold change of NHS-zym versus NHS **(C)**. Data are presented as mean ± standard deviation of three donors for primary GMVECs and nine independent experiments for control ciGMVECs. Experiments were performed with at least two replicates per condition. **(B)** Not significant (ns), ***P < 0.001, using two-way ANOVA followed by Tukey’s multiple comparisons test. **(C)** Not significant (ns), using unpaired t-test.

Human endothelial cell types from different origin have been used previously to model complement deposition *ex vivo*. We focused on studying complement activation on glomerular endothelial cells as these are the primary target in aHUS. Therefore, we first compared the performance of primary GMVECs with ciGMVECs as cellular models for complement activation *ex vivo*, by investigating the endothelial C5b-9 deposition upon incubation with NHS and zymosan-stimulated NHS. A baseline level of C5b-9 deposition was observed for both primary control GMVECs and control ciGMVECs upon incubation with NHS (**Figures 1A, B**). The amount of depositions significantly increased when the cells were incubated with NHS supplemented with zymosan to simulate an activated complement system (**Figures 1A, B**). Similar results were seen for the HMECs-1 (**Supplementary Figures 1A, B**), which is the most frequently described cellular model for aHUS (27–31), thereby further validating our results on the glomerular endothelial cell model.

To determine the discriminatory power between baseline C5b-9 deposition (NHS) and the C5b-9 level after incubation with activated complement (NHS-zymosan), the fold change of NHS-zymosan to NHS was calculated for primary GMVECs and ciGMVECs (**Figure 1C**). No significant difference was measured. We continued with validating ciGMVECs as a model for complement activation on the glomerular endothelial surface.

### 3.2 Human ciGMVECs as a model to adequately measure complement-mediated C5b-9 deposition

To determine if we could adequately and reliably measure complement-mediated C5b-9 deposition on the ciGMVECs, we visualized and quantified C5b-9 on the ciGMVECs upon incubation with different test sera in which complement was activated or blocked.

**Figure 2:**
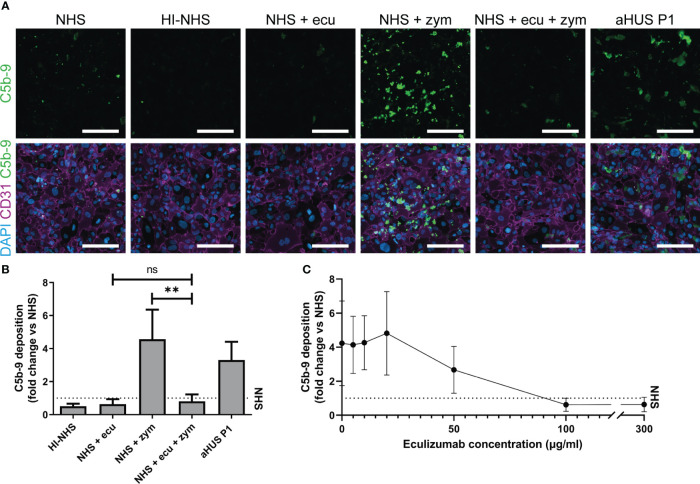
Human control conditionally immortalized glomerular microvascular endothelial cells (ciGMVECs) as model for complement deposition. **(A-C)** Control ciGMVECs were incubated with 33.3% serum in test medium for 2 hours. **(A)** Representative immunofluorescence images of C5b-9 (green), CD31 (purple) and cell nuclei with 4’, 6-diamidino-2-phenylindole (DAPI; blue). Incubation with normal human serum (NHS), heat-inactivated NHS (HI-NHS), NHS supplemented with 300 µg/ml eculizumab (ecu), NHS with zymosan (zym), NHS + ecu + zym and aHUS P1 serum. Scale bars: 200 µm. **(B)** Quantification of serum-induced C5b-9 deposition on control ciGMVECs. Data are presented as mean ± standard deviation of the fold change to NHS (dotted line) run in parallel of nine independent experiments, except for aHUS P1 (three independent experiments). Experiments were performed with at least two replicates per condition (single measurements for aHUS P1 serum). Not significant (ns), **P < 0.01, using one-way ANOVA followed by Bonferroni’s multiple comparisons test (aHUS P1 excluded for statistical analysis). **(C)** Eculizumab titration in NHS-zym. Data are presented as the mean ± standard deviation of the fold change to NHS (dotted line) run in parallel of three independent experiments performed with at least two replicates per condition.

First, we showed that incubation of ciGMVECs with either HI-NHS or NHS supplemented with eculizumab resulted in decreased C5b-9 depositions compared to NHS (**Figures 2A, B**). The increase in C5b-9 deposition after incubation with NHS-zymosan could also be inhibited when serum was first treated with eculizumab (**Figures 2A, B**). With flow cytometry as an alternative method for C5b-9 quantification we further confirmed our microscope imaging findings for ciGMVECs (**Supplementary Figure 2**). In line with the C5b-9 deposition, we showed that the deposition of C3 was increased upon incubation with NHS-zymosan (**Supplementary Figure 3**). Together, these results indicate that the generation of C5b-9 on the endothelial surface was complement-mediated. Incubation of ciGMVECs with serum of aHUS P1 resulted in increased C5b-9 deposition compared to NHS (**Figures 2A, B**). No significant differences in the C5b-9 deposition levels were observed between 1-, 2- or 3-hour serum incubations (**Supplementary Figure 4**). Two-hour incubation with serum was used as the standard method for further experiments.

Next, we titrated eculizumab in NHS-zymosan to assess how the cellular model reflects *in vivo* (fluid-phase) complement inhibition by eculizumab. The target serum level of eculizumab for aHUS patients for adequate blockage of complement is above 50-100 µg/ml (51, 52). In line with this, we observed maximal blockage of C5b-9 depositions on ciGMVECs with concentrations of 100 µg/ml and higher, and partial inhibition of C5b-9 formation with 50 µg/ml eculizumab (**Figure 2C**). Concentrations of 20 µg/ml and lower did not affect the C5b-9 depositions induced by activated serum.

### 3.3 C5b-9 deposition on human ciGMVECs in acute phase and remission

After validating ciGMVECs as a model to specifically measure complement-mediated C5b-9 depositions, we explored their usage for modeling aHUS by comparing aHUS patient samples obtained from different disease phases. Incubation of ciGMVECs with sera from P2, P3, P4, P5, and P6 obtained in the acute phase resulted in increased C5b-9 deposition levels, ranging from 1.37-7.69 fold the levels generated by NHS (**Table 2**; **Supplementary Figure 5**). In all serum samples, eculizumab levels were below the detection limit of 8 µg/ml.

**Table 2:** Complement C5b-9 deposition of atypical hemolytic uremic syndrome (aHUS) patient samples on human control conditionally immortalized glomerular microvascular endothelial cells (ciGMVECs).

Patient	Day of sampling	Eculizumab target >50-100 µg/ml	Disease phase	C5b-9 deposition (fold change vs NHS run in parallel)^d^
P2	0	<8	Acute phase (TMA + AKI)	2.84^e^
	13	206	Remission phase^a^	0.30
P3	0	<8	Acute phase (TMA + AKI)	3.97
	307	<8	Remission phase	0.76
P4	0	<8	Pre-relapse	0.68
	10	<8	Acute phase (TMA + AKI)^b^	1.37
	211	<8	Acute phase (TMA + AKI)^c^	7.69
	344	<8	Remission phase	0.43
P5	0	<8	Acute phase (TMA + AKI)	2.17
	15	134	Remission phase^a^	0.59
P6	0	<8	Acute phase (TMA + AKI)	5.85

a Eculizumab level was above therapeutic target, suggesting adequate complement blockage.

b Lactate dehydrogenase (LDH) 1450 U/l, thrombocytes 21×10^9^/l, urine protein-to-creatinine ratio (UPCR) 1.18 g/10mmol, and serum creatinine 130 µmol/l.

c LDH 612 U/l, thrombocytes 65×10^9^/l, UPCR 0.37 g/10mmol, and serum creatinine 128 µmol/l.

d Patient samples: mean of two replicates of a single experiment. NHS: mean of at least three replicates of a single experiment.

e No duplicate.

AKI: acute kidney injury, NHS: normal human serum, TMA: thrombotic microangiopathy.

Surprisingly, the sample of P4 obtained during evident hematological TMA and AKI (d10; **Table 2**) showed only slightly increased C5b-9 deposition compared to NHS. On the contrary, a second acute phase sample (d211; **Table 2**), showed higher C5b-9 deposition, despite the clinically less pronounced TMA. Of note, both relapses were triggered by a viral infection.

The remission samples of P2 and P5 with an eculizumab level above the therapeutic target suggesting adequate complement blockage, showed normalized C5b-9 depositions (**Table 2**; **Supplementary Figure 5**). The remission sera from P3 and P4, obtained while in remission after eculizumab withdrawal, also resulted in normalized endothelial C5b-9 deposition levels (**Table 2**; **Supplementary Figure 2**).

### 3.4 Monitoring C5b-9 deposition in an aHUS patient during eculizumab treatment on control and patient ciGMVECs

**Figure 3:**
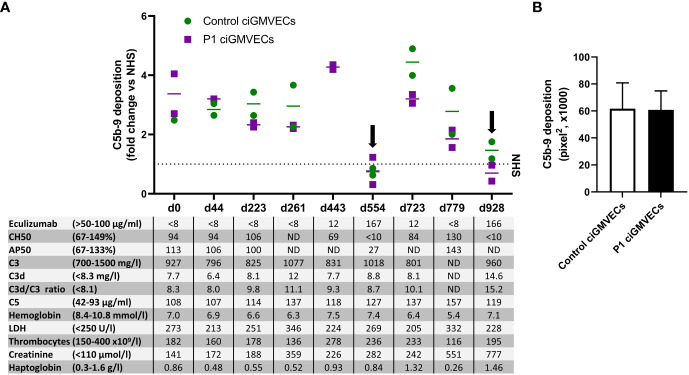
Monitoring C5b-9 deposition by aHUS P1 during follow-up on control conditionally immortalized glomerular microvascular endothelial cells (ciGMVECs) and P1 ciGMVECs. **(A, B)** ciGMVECs derived from a control (green) or aHUS P1 (purple) were incubated with 33.3% serum in test medium for 2 hours. aHUS P1 sera were obtained during follow-up, indicated by days (d). **(A)** Data are presented as mean of two measurements (bars) or single measurements (squares and circles), all expressed in fold change compared to normal human serum (NHS) run in parallel (dotted line) performed in a single experiment for each cell line. The table inset shows the corresponding laboratory parameters for complement activation and TMA per test sample. Reference values of laboratory parameters are indicated between parentheses. Target values for adequate complement blockage by eculizumab are eculizumab >50 µg/ml, CH50 <10%, and AP50 <30%. Samples with adequate serum eculizumab levels are indicated with an arrow. LDH: lactate dehydrogenase, ND: not determined. **(B)** C5b-9 deposition on control ciGMVECs and P1 ciGMVECs after incubation with NHS. Data are presented as mean ± standard deviation of seven independent experiments for P1 ciGMVECs and nine independent experiments for control ciGMVECs. Not significant, using unpaired t-test. Experiments were performed with at least two replicates per condition.

P1 developed a severe form of aHUS from the age of 5 months, which is described in detail in the materials and methods and by Bouwmeester et al. (36). We thoroughly monitored P1 during his chronic kidney failure and eculizumab treatment with various complement (activation) and TMA markers in the blood and studied if these parameters correlated with endothelial C5b-9 depositions. Interestingly, all samples of P1 in which the eculizumab concentrations were below the therapeutic target (<50 µg/ml) showed increased C5b-9 deposition on ciGMVECs (**Figure 3A**), irrespective of the clinical disease phase the samples were taken from. Thus, also samples taken when the patient did not show active signs of TMA showed increased cellular C5b-9 deposition. During adequate complement blockage, indicated by serum eculizumab levels above 50-100 µg/ml and a CH50 <10%, C5b-9 deposition was comparable or below NHS level (**Figure 3A**).

As this patient was not a “typical” aHUS patient because of ongoing decline in kidney function (described by Bouwmeester et al. (36)) and high C5b-9 deposition levels despite “quiescent” TMA markers, we hypothesized the glomerular endothelium of this patient might be more susceptible to complement activation and/or complement-mediated damage. When this patient received a kidney transplant, we were able to isolate GMVECs from the tissue of the removed native kidney from this patient to test this hypothesis. Incubation of the same series of serum samples obtained during follow-up with the patient’s own ciGMVECs (P1 ciGMVECs) resulted in C5b-9 deposition that was similar to the deposition on control ciGMVECs (**Figure 3A**). Also, the patient cells were similarly susceptible to C5b-9 deposition induced by NHS (**Figure 3B**). The C5b-9 levels were not higher for P1 ciGMVECs compared to control ciGMVECs based on the fold change compared to NHS after incubation with different test sera (**Supplementary Figure 6**).

## 4 Discussion

aHUS is a complex disease characterized by dysregulation of the AP, primarily caused by genetic variants in genes encoding complement components and/or by autoantibodies. Uncontrolled complement activity in aHUS causes AP activation and C5b-9 deposition on the GMVECs in the glomerulus. This contributes to endothelial cell activation and endothelial damage as well as a prothrombotic phenotype, leading to decreased kidney function. In this study, we showed that human control ciGMVECs and aHUS patient ciGMVECs can be used to adequately measure and model complement C5b-9 deposition on endothelial cells by different test sera and aHUS patient sera. Furthermore, aHUS patient ciGMVECs were incubated with patient’s own serum samples.

The glomerulus is the main target of the complement-mediated damage in aHUS, suggesting a specific susceptibility of these cells to complement activation (24, 25, 53). Endothelial cells from different origin have different expression profiles of complement regulators and complement proteins, which may lead to different susceptibility for complement activation (in resting or activated state) (24, 25, 54, 55). Furthermore, glomerular endothelial cells may have different adaptive mechanisms for certain TMA triggers such as heme. For example, May et al. (56) showed a higher susceptibility for complement deposition of glomerular endothelial cells compared to other cell types upon heme stimulation. Thus, to reflect the physiological context as close as possible, glomerular endothelial cells were chosen as the preferred cell type for modeling aHUS *ex vivo*.

First, we showed that no significant differences were observed between primary GMVECs and control ciGMVECs with respect to their susceptibility for C5b-9 deposition by NHS and NHS-zymosan. Even though human primary GMVECs might reflect the *in vivo* situation better than ciGMVECs, primary GMVECs have several limitations (also reviewed in Meuleman et al. (53)). First, human primary GMVECs have limited availability and their isolation and culture is labor-intensive and requires great expertise. Second, these cells have a limited lifespan. Thus, multiple individual donors are needed resulting in differences in genetic background, and thereby inter-experimental variability. ciGMVECs do not have these practical limitations and are more suitable for standardized testing.

We showed that human ciGMVECs are a reliable and robust model for complement-mediated C5b-9 deposition, visualized and quantified with immunofluorescence microscopy. NHS-zymosan, which simulates an overactive complement system, resulted in increased C5b-9 deposition compared to NHS. On the other hand, inhibition of the complement system in NHS with eculizumab resulted in a dose-dependent decrease in C5b-9 deposition. Our results were in line with the recommended treatment target of eculizumab *in vivo*. Zymosan is obtained out of the cell wall of *Saccharomyces cerevisiae* and activates the complement system in serum via the AP by promoting rapid C3 cleavage (57, 58). However, zymosan might also activate endothelial cells as it is recognized by Toll-like receptors (59, 60). We could not identify to what extent cellular activation may have contributed to increased C5b-9 deposition.

In all acute phase samples of aHUS patients, increased C5b-9 deposition was observed compared to pooled NHS on (unstimulated) ciGMVECs. However, the most evident relapse for P4 (d10) did not show the highest C5b-9 deposition level in the assay compared to the other relapse (d211), which might indicate that the amount of C5b-9 deposition may not exactly reflect the disease state or that there may be a delay between clinical presentation and the complement effect on the endothelium. The assay detected normalized endothelial C5b-9 deposition levels in patients samples (P2-P5) in remission (either with or without adequate complement blockage by eculizumab).

Interestingly, all sera of P1, except the samples with eculizumab levels above target, induced elevated endothelial C5b-9 depositions, even though samples were obtained from disease phases without clinical or laboratory signs of TMA or complement activation present. The same effect was seen when the samples were tested on the patient’s own ciGMVECs. This suggests that even though P1 showed a highly active complement deposition profile and ongoing decline in kidney function, the cells were not intrinsically, in resting state, more susceptible to complement activation (neither by patient serum nor by NHS). The cause of the high C5b-9 depositions in this patient with chronic kidney failure remains therefore unknown. Further studies are required to identify if patients with chronic kidney failure, either with or without aHUS, have increased complement activation.

Roumenina et al. (34, 61) and Frimat et al. (33) were the first to describe complement deposition on human glomerular endothelial cells (ciGMVECs). The authors described C3 deposition as mean readout for complement activation, which was primarily quantified with flow cytometry. Frimat et al. observed increased complement deposition in several aHUS patients versus control samples on resting cells, but for identifying all aHUS patient samples stimulation of the cells with heme was required (33). In Roumenina et al. (34), TNFα/IFNγ activation was used to discriminate healthy controls from aHUS patients with a C3 mutation. The authors did not describe or compare the disease phases of the aHUS patients. We did not need to pre-activate our cells to distinguish increased complement deposition in aHUS patients from controls. Thereby, our results are in line with groups working with HMECs-1 showing that the aHUS samples in the acute phase can be identified on resting cells (27, 31). This assay was first described by Noris et al. (27) and followed-up by Galbusera et al. (31) as an assay with the diagnostic aim of monitoring and individualizing eculizumab therapy and predicting relapses. Timmermans et al. (28) used the assay for showing complement depositions in hypertension-associated TMA, and Palomo et al. (29) published an adapted methodology with activated plasma samples and analyzed pre-eclampsia and HELLP (a pregnancy complication characterized by hemolysis, elevated liver enzymes, and low platelet count) patient samples in addition to aHUS samples. Some of these groups also showed increased C5b-9 deposition on activated cells after incubation with serum from unaffected mutation carriers and/or patients in the remission phase (27, 28, 34). We did not test such conditions as this was beyond the scope of our study.

To our knowledge, we are the first to show that unique aHUS patient GMVECs can be obtained, conditionally immortalized, and used as a cell line for experimental testing. This approach offers great promise for further research into the pathophysiology of aHUS, since it is now possible to align the endothelial cells of a patient with its own serum samples. In our case, the aHUS patient had a *CFH-CFHR1* hybrid gene, but it will also be very valuable to use this tool to generate cell lines from aHUS patients with mutations in membrane-bound regulators or from patients without confirmed complement mutations. This allows to investigate the effect of differences in endothelial cell characteristics on complement activation and susceptibility. After all, it remains unknown why some complement mutation carriers develop aHUS and others do not and if there are endothelial susceptibility factors contributing to aHUS in patients without proven genetic aberrations.

Our imaging strategy allows visualization of large areas of the well in one time in a reliable and fast way, even though the resolution may be lower compared to traditional confocal microscopy. In this study, the number of aHUS patients was still limited. Testing more aHUS patients with different genetic background during follow-up (and treatment) will help us to further understand aHUS pathophysiology. Nonetheless, it is important to emphasize that the *ex vivo* complement activity on endothelial cells cannot fully reflect the *in vivo* situation. For example, it has been suggested that the vascular heterogeneity of the glycomatrix is involved in glomerular diseases (24, 62). In addition, FH and properdin can interact with glycosaminoglycan heparan sulfate produced by endothelial cells (63). As these static 2D models do not fully reflect the glomerular organization, 3D models involving multiple cell types combined with flow conditions might be of potential to mimic the *in vivo* situation more closely.

In conclusion, we showed that human control ciGMVECs and aHUS patient ciGMVECs can be used to study and quantify endothelial C5b-9 complement deposition after serum incubation. Furthermore, by using human ciGMVECs, the described protocol is a promising tool to study complement pathophysiological mechanisms in the kidney, in particular the pathophysiology of aHUS. In addition, the model may be used for *in vitro* testing of novel complement therapeutics. The use of aHUS patient-derived (genetically altered) ciGMVECs might be of help to further explore the susceptibility of the endothelium to the disease under various circumstances.

## Data availability statement

The original contributions presented in the study are included in the article/**Supplementary Material**. Further inquiries can be directed to the corresponding author.

## Ethics statement

The studies involving humans were approved by Ethics Committee of Oost-Nederland (registration number of the CUREiHUS study: NL52817.091.15). The studies were conducted in accordance with the local legislation and institutional requirements. Written informed consent for participation in this study was provided by the participants’ legal guardians/next of kin. Written informed consent was obtained from the individual(s), and minor(s)’ legal guardian/next of kin, for the publication of any potentially identifiable images or data included in this article.

## Author contributions

Concept of the study: NK and LH; Design of the experiments: KS, LB, TV, NK, and LH; Experimental work: KS, LB, TV, and ND; Data interpretation: KS, LB, ND, MM, RB, NK, and LH; Collection and characterization of clinical samples: KS, LB, RB, JW, ED, AZ NK, and LH; Resources: NK and LH; Supervision: LB, MM, NK, and LH; Project administration: LH; Manuscript writing – original draft: KS; Manuscript writing – reviewing and editing: LB, MM, JW, ED, AZ, NK, LH, and RB. All authors contributed to the article and approved the submitted version.
