# Supplementary material for: Modeling complement activation on human glomerular microvascular endothelial cells
Source: Front Immunol. 2023 Oct 25;14:1206409. doi: 10.3389/fimmu.2023.1206409 (PMC10634509; doi:10.3389/fimmu.2023.1206409)
Supplement: Supplementary file 1 [file DataSheet_1.docx]

Supplementary Material

# Supplementary Figures


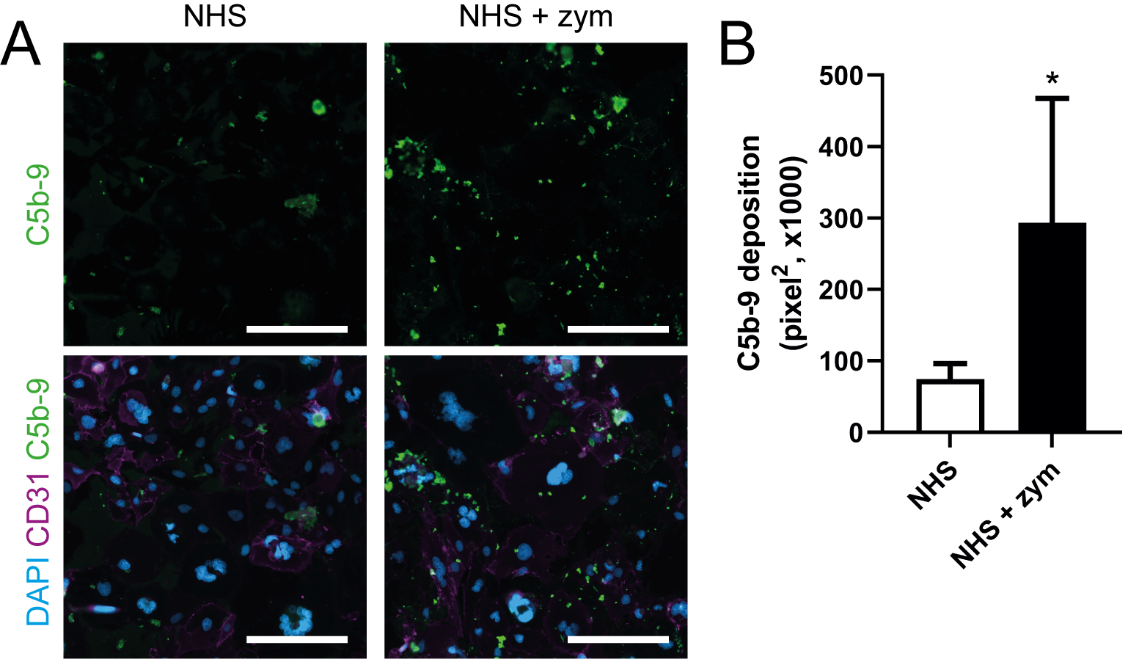


**Supplementary Figure 1. Serum-induced C5b-9 deposition on human dermal microvascular endothelial cells 1 (HMECs-1).** HMECs-1 were incubated with 33.3% serum in test medium for 2 hours. (A) Representative immunofluorescence images of C5b-9 (green), CD31 (purple) and cell nuclei with 4', 6-diamidino-2-phenylindole (DAPI; blue) after incubation with normal human serum (NHS) and NHS-zymosan (zym). Scale bar: 200 µm. (B) Quantification of C5b-9 deposition in pixel^2^ after serum incubation with NHS and NHS-zym. Data are presented as mean ± standard deviation of five individual experiments. * P<0.05, using unpaired t-test.


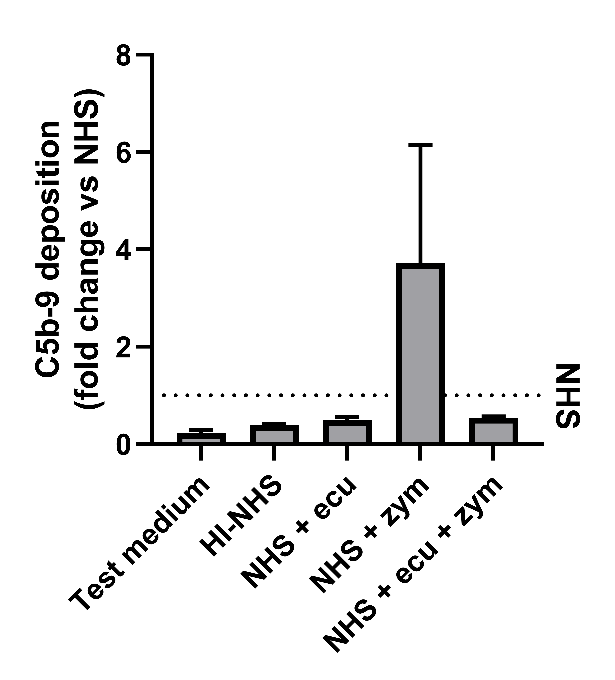


**Supplementary Figure 2. Quantification of C5b-9 deposition on human control conditionally immortalized glomerular microvascular endothelial cells (ciGMVECs) with flow cytometry.** ciGMVECs were incubated with 10% serum in test medium for 1 hour. C5b-9 deposition in fold change compared to normal human serum (NHS) run in parallel after incubation with different test conditions. Data are presented as mean ± standard deviation of three individual experiments. Ecu: eculizumab, HI: heat-inactivated, zym: zymosan.

**
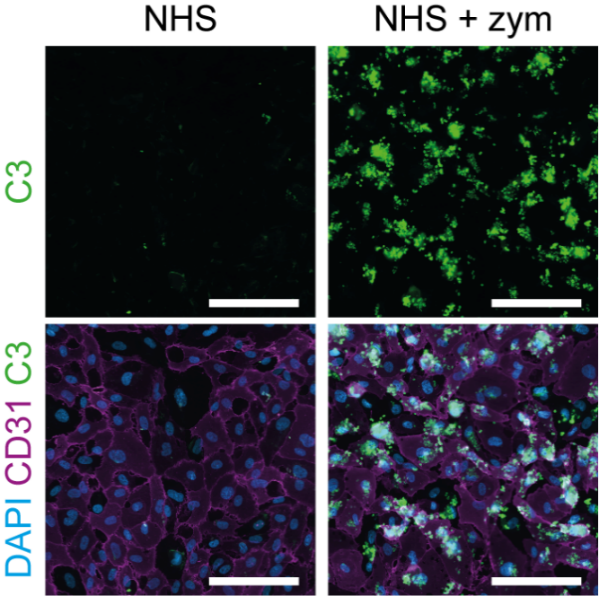
**

**Supplementary Figure 3. Increased C3 deposition after incubation with normal human serum (NHS) with zymosan (zym).** Human control conditionally immortalized glomerular microvascular endothelial cells (ciGMVECs) were incubated with 33.3% serum in test medium for 2 hours. Representative immunofluorescence images (three independent experiments) of C3 (green), CD31 (purple) and cell nuclei with 4’, 6-diamidino-2-phenylindole (DAPI; blue). Scale bars: 200 µm.


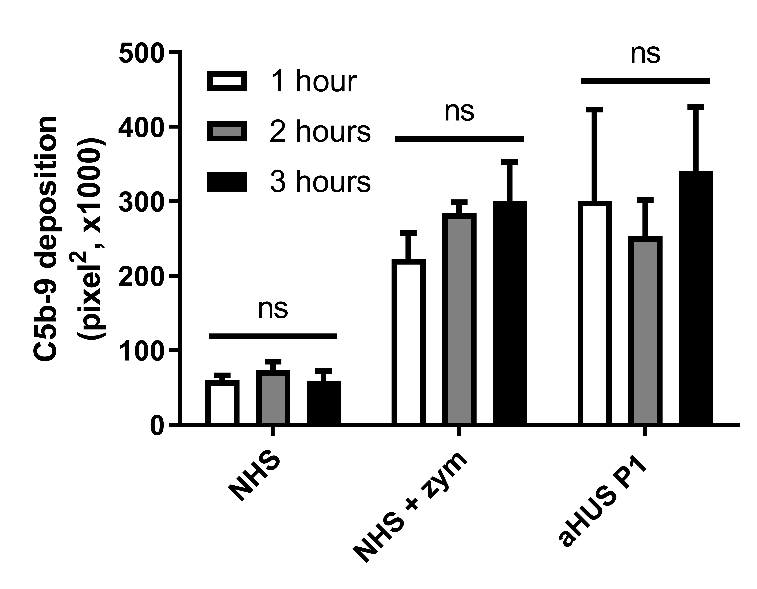


**Supplementary Figure 4. Effect of incubation time on C5b-9 deposition.** Human control conditionally immortalized glomerular microvascular endothelial cells (ciGMVECs) were incubated with 33.3% serum in test medium. Data are presented as mean ± standard deviation of at least three (n = 3-5) independent experiments per condition. Not significant (ns), using two-way ANOVA followed by Bonferroni’s multiple comparison test. NHS: normal human serum, zym: zymosan.


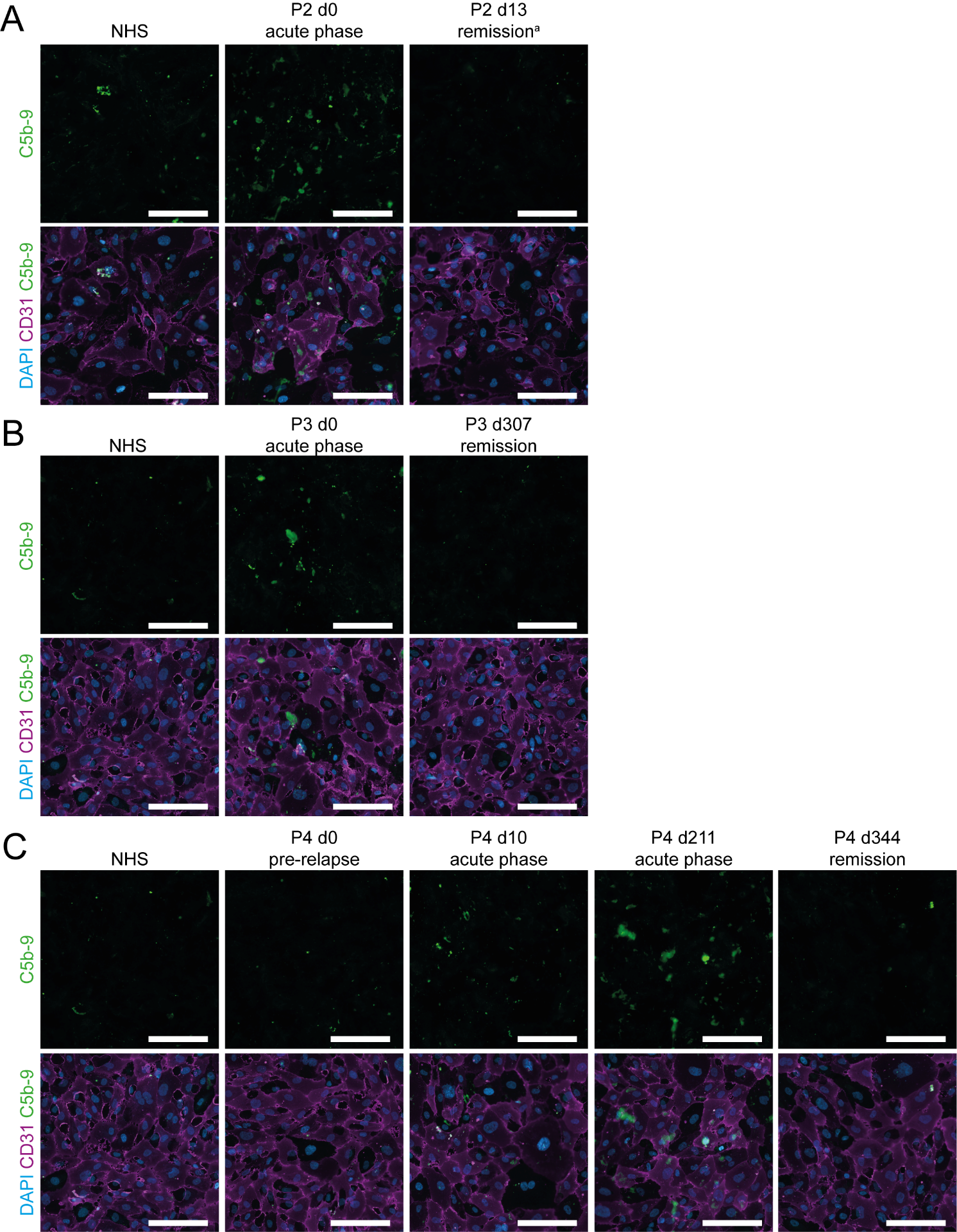


**
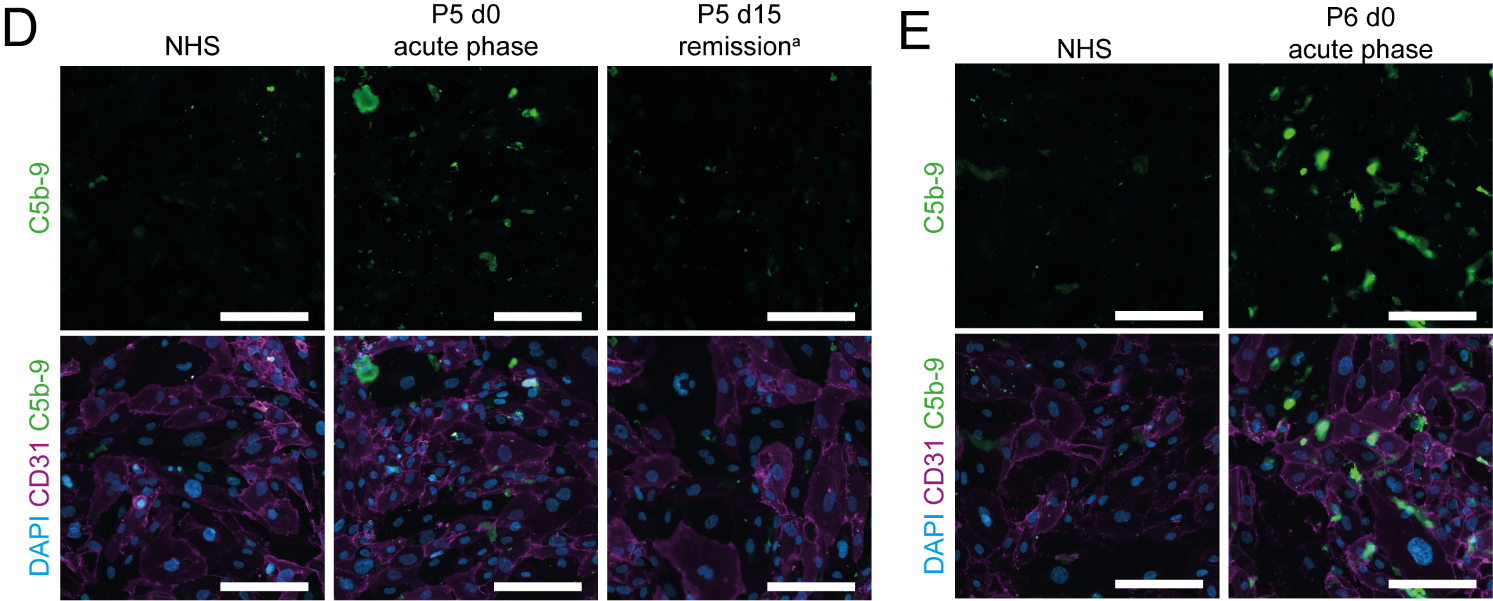
**

**Supplementary Figure 5. Complement C5b-9 deposition of aHUS patient samples on human control conditionally immortalized glomerular microvascular endothelial cells (ciGMVECs).** ciGMVECs were incubated with 33.3% serum in test medium for 2 hours. Representative immunofluorescence images of C5b-9 (green), CD31 (purple) and cell nuclei with 4', 6-diamidino-2-phenylindole (DAPI; blue) from two replicates for patient serum and four replicates for normal human serum (NHS). Scale bars: 200 µm. d: day. ^a^ Eculizumab level above therapeutic target.


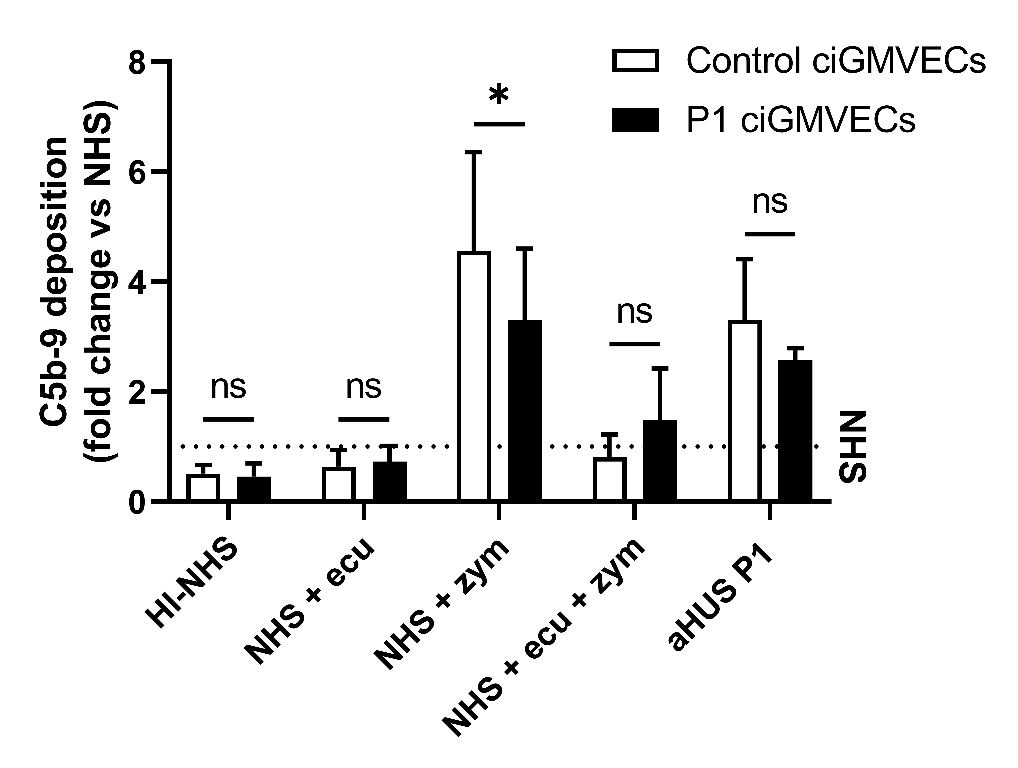


**Supplementary Figure 6. Serum-induced C5b-9 deposition on human control conditionally immortalized glomerular microvascular endothelial cells (ciGMVECs) and P1 ciGMVECs.** Control ciGMVECs and P1 ciGMVECs were incubated with 33.3% serum in test medium for 2 hours. C5b-9 deposition in fold change compared to normal human serum (NHS) run in parallel after incubation with different test conditions. Data are presented as mean ± standard deviation of nine independent experiments for control ciGMVECs and seven independent experiments for P1 ciGMVECs, except for aHUS P1 serum (three independent experiment for each cell line). Experiments were performed with at least two replicates per condition (single measurements for aHUS P1 serum). Not significant (ns), * P<0.05, using two-way ANOVA followed by Bonferroni’s multiple comparisons test. Ecu: eculizumab, HI: heat-inactivated, zym: zymosan
